# Adult head and neck rhabdomyosarcoma: radiotherapy- based treatment, outcomes, and predictors of survival

**DOI:** 10.1186/s12885-024-12079-y

**Published:** 2024-03-14

**Authors:** Dan Zhao, Fang Zhou, Weixin Liu, Zhou Huang, Xiaolong Xu, Baomin Zheng, Changqing Liu, Chujie Bai, Jiayong Liu, Yan Sun, Weihu Wang, Shaowen Xiao

**Affiliations:** 1 Key Laboratory of Carcinogenesis and Translational Research (Ministry of Education/Beijing), Department of Radiation Oncology, Peking University Cancer Hospital & Institute, Peking University, 100142 Beijing, P.R. China; 2 Department of Radiotherapy, The Affiliated Yantai Yuhuangding Hospital of Qingdao University, Shandong, P.R. China; 3 Key Laboratory of Carcinogenesis and Translational Research (Ministry of Education/Beijing), Department of Bone and Soft Tissue Oncology, Peking University Cancer Hospital & Institute, Peking University, 100142 Beijing, P.R. China

**Keywords:** Head and neck, Adult, Rhabdomyosarcoma, Prognosis, Radiotherapy

## Abstract

**Background:**

Adult head and neck rhabdomyosarcoma (HNRMS) is an exceptionally rare malignancy, and there is a paucity of data and research dedicated to understanding its characteristics and management in adult populations. This study aimed to assess the outcomes and identify survival predictors in adult HNRMS.

**Methods:**

We retrospectively evaluated 42 adult patients (> 16 years) with HNRMS who received radiotherapy (RT)-based treatment at our institute between 2008 and 2022. We analysed the clinical characteristics and prognosis of these patients, including the locoregional recurrence-free survival (LRFS), progression-free survival (PFS), and overall survival (OS), using the Kaplan–Meier method. The chi-square and Fisher’s exact tests were used to analyse differences between groups for dichotomous and categorical variables, respectively. Survival rates were calculated using the Kaplan–Meier method. Prognostic variables were assessed through univariate Cox analyses.

**Results:**

The median patient age was 28 years (range, 16–82 years). Alveolar RMS was the most common histological type, observed in 21 patients (50.0%), followed by embryonal in 16 patients (38.1%). The anatomic sites of origin were orbital in one (2.4%), parameningeal in 26 (61.9%), and non-orbital/non-parameningeal in 15 (35.7%) patients. Nineteen patients (45.2%) had regional lymph node metastasis, and five patients (11.9%) presented with distant metastatic disease. Distant metastasis (*n* = 17) was the primary cause of treatment failure. At a median follow-up of 47.0 months, the 5-year LRFS, PFS, and OS rates were 69.0%, 39.7%, and 41.0%, respectively. Univariate analysis revealed that tumour size, lymph node involvement, and the local treatment pattern (surgery and RT vs. RT alone) were significant predictors of survival.

**Conclusions:**

The main failure pattern in patients with HNRMS receiving RT-based treatment was distant metastasis. Tumour size > 5 cm and lymph node involvement were predictors of worse LRFS. Multimodality local treatment, combining surgery and RT, is effective and provides survival benefits.

## Background

Rhabdomyosarcomas (RMS) are more common among children and adolescents but are exceedingly rare in adults. RMS accounts for 2–5% of all soft tissue sarcomas (STS), whereas STS accounts for less than 1% of adult malignancies [1]. Among adults with RMS, 9% present with a primary disease of the head and neck (HNRMS) [2].

RMS has three histological subtypes: embryonal (including botryoid and spindle cell variants), alveolar (including a solid variant), and pleomorphic [3, 4]. The distribution of RMS histological subtypes differs between paediatric and adult populations: embryonal and alveolar variants are more common in children and adolescents, whereas the pleomorphic variant is more common in adults [5]. In the paediatric population, the prognosis varies dramatically between these histological subtypes, with 5-year overall survival (OS) rates of 82.0% for embryonal and 53.0% for alveolar HNRMS [6]. Due to the extremely low incidence rate and lack of large-scale clinical research, no consensus currently exists on whether histological type also affects the prognosis of adult HNRMS.

In addition to the histological subtypes, the anatomic location of HNRMS also influences risk stratification and treatment approaches. HNRMS is classified into three categories based on these anatomic locations: orbital, parameningeal (paranasal sinus, nasal cavity, nasopharynx, skull base, mastoid, middle ear, infratemporal, and pterygopalatine fossae), and non-orbital/non-parameningeal [7]. Primary sites for RMS are broadly classified as favourable or unfavourable, with parameningeal locations defined as unfavourable sites in HNRMS [8].

Based on the Intergroup Rhabdomyosarcoma Study Group (IRSG) protocols, recommended treatments for childhood RMS include gross total resection with preservation of function, systemic chemotherapy, and radiotherapy (RT). With this multimodality treatment, the survival of children with RMS has improved significantly over the past 30 years, with a 5-year OS rate of 70.0–80.0% [9, 10]. Although the treatment experiences of childhood RMS have been widely extrapolated to adults, the outcome of adult RMS remains unsatisfactory, with a 5-year OS rate of 20.0–40.0% [9, 11–13]. These disparities in outcomes may reflect differences in pathogenesis, raising doubts regarding whether chemotherapy should be used as the mainstay of therapy in adult RMS as it is in children’s treatment.

Given the rarity and limited clinical experience (mostly from single-institution retrospective studies), no standard treatment for adult HNRMS exists [14, 15]. Similar to other types of STS, RT is often considered the local treatment of choice for HNRMS due to the high morbidity associated with extensive surgery [16]. Owing to anatomical limitations, extended radical resection is difficult to perform; therefore, RT is almost always utilised for HNRMS, regardless of the degree of resection. Regarding radiation therapy, the widespread application of intensity-modulated radiation therapy (IMRT) offers a more precise treatment with lower toxicity [17, 18].

Here, we report our experience with 42 adult patients with HNRMS treated at our institute, the Radiation oncology department of a large tertiary cancer centre. This study aims to provide clinical insights into this rare disease and to identify the clinicopathologic and treatment-related predictors of HNRMS in adults.

## Patients and methods

We performed a retrospective analysis of 42 adult patients with HNRMS (> 16 years of age). The patients underwent radiation therapy at our institute between June 2008 and June 2022. Pertinent patient data, including baseline characteristics, staging, histologic type, surgical margin, mode of therapy, and outcomes, were analysed. We retrospectively restaged patients using the IRSG staging system and the American Joint Committee on Cancer (AJCC) Staging System for head and neck STS (8th ed, 2017).

**Fig. 1:**
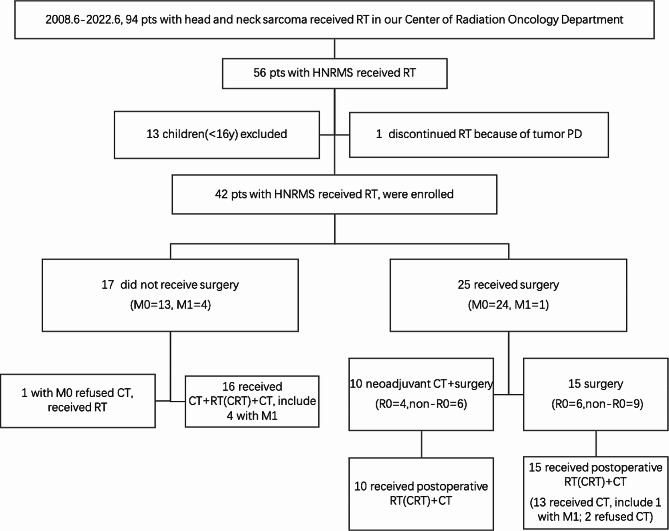
The flow diagram of this study

The flow diagram of treatment is shown in Fig. 1. A multimodality treatment plan (surgery, RT, and chemotherapy) was individually designed for each patient. Treatment recommendations and decisions were based on comprehensive considerations.

If surgery was feasible, the tumour was resected as much as possible to obtain acceptable cosmetic and functional outcomes. An advantage of upfront surgical resection is accurate pathological classification and direct risk stratification. The postsurgical IRSG grouping was based on the degree of resection completed, which was useful in evaluating the role of RT. R0 was defined as a resection with free margins /no residual disease, both macroscopic and microscopic. R1 was defined as a resection with microscopic residual disease, and R2 was defined as a resection with gross residual disease. However, a negative margin is usually not feasible in HNRMS, particularly for parameningeal tumours, and preoperative chemotherapy is recommended for these patients.

Chemotherapy consisted mostly of a combination of 2–3 agents, including vincristine, doxorubicin, epirubicin, cyclophosphamide, ifosfamide, cisplatin, and etoposide. Different combinations and doses of these agents were modified according to patients’ performance status and risk groups. Chemotherapy was administered preoperatively, postoperatively, both pre-and postoperatively (where applicable), during radical chemoradiotherapy, or first-line chemotherapy. Responses to chemotherapy were assessed by investigators according to the Response Evaluation Criteria in Solid Tumors (RECIST) version 1.1 using computed tomography (CT) or magnetic resonance imaging (MRI) findings. Responses could not be assessed in 15 patients for the following reasons: complete resection of the localised disease at the initiation of treatment (12 patients) and refusal to undergo chemotherapy (3 patients).

RT was administered using IMRT in three different settings: definitive, postoperative, and primary tumour RT for patients with distant metastasis. Target delineation involved the following: GTV (gross tumour volume)/GTVtb (gross tumour volume of tumour bed)– This encompasses the primary tumour area and positive lymph nodes or postoperative tumour bed areas. This was mainly determined based on imaging findings with CT or MRI (or both); CTV (clinical target volume)– This is an expansion of 2–3 cm around the GTV. It also includes routes of spread at the external margin of GTV (bone, fascia, and other anatomical structures acting as natural barriers can form natural boundaries of the CTV, which can be ≤ 1 cm). Additionally, because the regional nodal involvement rates of HNRMS were as high as 42.3-50.0% in previous literatures [2, 11], elective nodal irradiation (ENI)was recommended; PTV (planning target volume)– This is an expansion of 3 mm from the CTV. Dose prescriptions are as follows: definitive RT - GTV (66–70 Gy, 2–2.2 Gy/f); adjuvant RT - GTV/GTVtb (R0/R1, 60–66 Gy, 2–2.2 Gy/f; R2, 66–70 Gy, 2–2.2 Gy/f); CTV dose was given in the range of 50–54 Gy in 1.8–2 Gy per fraction. After treatment, patients were followed up at 3- to 4-month intervals for the first 2 years, 4- to 6-month intervals for the next 3 years, and annually thereafter.

### Statistical analysis

Time to locoregional recurrence or distant relapse was calculated from the first day of treatment. Survival was measured from the time of diagnosis to the time of death for any reason or last follow-up.

SPSS (version 24.0) was used for statistical analysis. The chi-square and Fisher’s exact tests were used to analyse differences between groups for dichotomous and categorical variables, respectively. Survival rates were calculated using the Kaplan–Meier method. Prognostic variables, including sex, age, tumour size, primary site, lymph node involvement, staging, histologic type, and mode of therapy, were analysed by univariate Cox analyses using log-rank statistics. Statistical significance was defined as a two-sided *P*-value < 0.05.

## Results

### Patient characteristics

The clinicopathological and treatment characteristics of the 42 patients included in this study are presented in Table 1. Sixteen patients (38.1%) had favourable prognostic sites, and 26 patients (61.9%) had unfavourable prognostic sites. The primary tumour size ranged from 2 to 9 cm (median, 5 cm). Female patients were more likely to experience lymph node metastasis than male patients (*P* = 0.034). There were no statistically significant differences in patient age, histology, primary tumour location, or tumour size according to lymph node status (Table 2). The number of embryonal, alveolar, pleomorphic and NOS histology was 10, 9, 2 and 2 for male, and 6, 12, 1, and 0 for female, respectively.

**Table 1:** Baseline of patients’ characteristics (*n* = 42)

Characteristics	n	%
Age, median (range)	28 year (16–82 year)	
<28yr	20	47.6
≥28yr	22	52.4
Gender		
Male	23	54.8
Female	19	45.2
Histology		
Embryonal	16	38.1
Alveolar	21	50.0
Pleomorphic	3	7.1
NOS	2	4.8
Primary site		
Orbital	1	2.4
Parameningeal	26	61.9
Non-orbital/non-parameningeal	15	35.7
Primary tumor size		
≤ 5 cm	19	45.2
> 5 cm	23	54.8
Nodal status		
N0	23	54.8
N1	19	45.2
Disease status		
Localized (N0M0)	21	50.0
Regional (N1M0)	16	38.1
Distant (M1)	5	11.9
IRS pretreatment staging		
Stage I	15	35.7
Stage II	2	4.8
Stage III	20	47.6
Stage IV	5	11.9
IRS postsurgical grouping		
Group I	9	21.4
Group II	11	26.2
Group III	17	40.5
Group IV	5	11.9
Surgery margins		
Negative (R0)	10	40.0
Positive (R1/2)	15	60.0
Treatment patterns		
Surgery + Chemotherapy + RT	23	54.7
Surgery + RT	2	4.8
Chemotherapy + RT	16	38.1
RT	1	2.4

NOS, not otherwise specified; Intergroup Rhabdomyosarcoma Study, IRS; RT, radiotherapy

**Table 2:** Distribution of patients’ characteristics by lymph node involvements

Characteristics	Lymph node involvements		
	N0(n)	N1(n)	*P* value
Age			0.976
<28 yr	11	9	
≥ 28 yr	12	10	
Gender			0.034
Male	16	7	
Female	7	12	
Histology			0.477
Embryonal	8	8	
Alveolar	11	10	
Pleomorphic + NOS	4	1	
Primary site			0.429
Parameningeal	13	13	
Non-Parameningeal	10	6	
Primary tumor size			0.711
≤ 5 cm	11	8	
> 5 cm	12	11	

NOS, not otherwise specified

### Treatment

All patients received an RT-based multimodality treatment, except for one 82-year-old patient who received RT alone due to intolerance to surgery and chemotherapy. Twenty-five patients (59.5%) underwent surgical excision of their primary tumours, of whom 10 underwent radical resections with R0 margins, 10 underwent resections with R1 margins, and five underwent subtotal excision with R2 margins. The remaining 17 patients underwent biopsy only. ENI is recommended at our institution for patients with HNRMS with a likelihood of cervical lymph node metastasis. Systemic chemotherapy was administered to 39 of the 42 patients (92.9%). The details are presented in Table 3. Responses were evaluated in 27 patients receiving systemic chemotherapy. The overall response rate (ORR; complete or partial response) was 51.8%, and the disease control rate (DCR; complete, partial, or stable response) was 96.3%. Definitive RT was administered to 13 patients, postoperative RT to 24 patients, and primary site RT to 5 patients with distant metastasis at presentation.

**Table 3:** Chemotherapy type, chemoradiotherapy type (*n* = 39), and response to Chemotherapy (*n* = 27)

	Disease status at diagnosis		All patients, n (%)
	Localized/locoregional (n)	Metastatic (n)	
Chemotherapy type			
postoperative only	12	0	12 (30.8)
both pre-and postoperative	10	0	10 (25.6)
first-line chemotherapy	0	5	5 (12.8)
radical chemoradiotherapy	12	0	12 (30.8)
Chemoradiotherapy type			
Sequential chemoradiation (SCRT)	25	2	27 (69.2)
Concurrent chemoradiation (CCRT)	9	3	12 (30.8)
Response to chemotherapy			
CR	1	0	1 (3.7)
PR	9	4	13 (48.1)
SD	10	2	12 (44.5)
PD	1	0	1 (3.7)

CR, complete response; PR, partial response; SD, stable disease; PD, progressive disease

### Disease outcome and patient survival

The followup timeframe ranged from 18.6 to 75.5 months, with a median follow-up period of 47.0 months. During follow-up, it was found that the disease recurred in 21 (50.0%) patients, and 17 (40.5%) patients died. The sites of first recurrence were local in four patients, metastatic in 13, local + metastatic in two, and regional + metastatic in two. Bone was the most common site of distant metastasis (50.0%), followed by the lungs (25.0%).

**Fig. 2:**
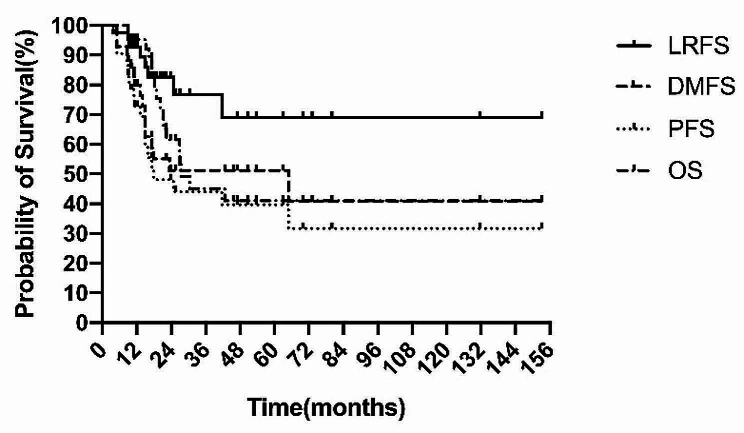
LRFS, DMFS, PFS, and OS curves for 42 patients with HNRMS Abbreviations: LRFS, locoregional recurrence-free survival; PFS, progression-free survival; DMFS, distant metastasis-free survival; OS, overall survival

**Fig. 3:**
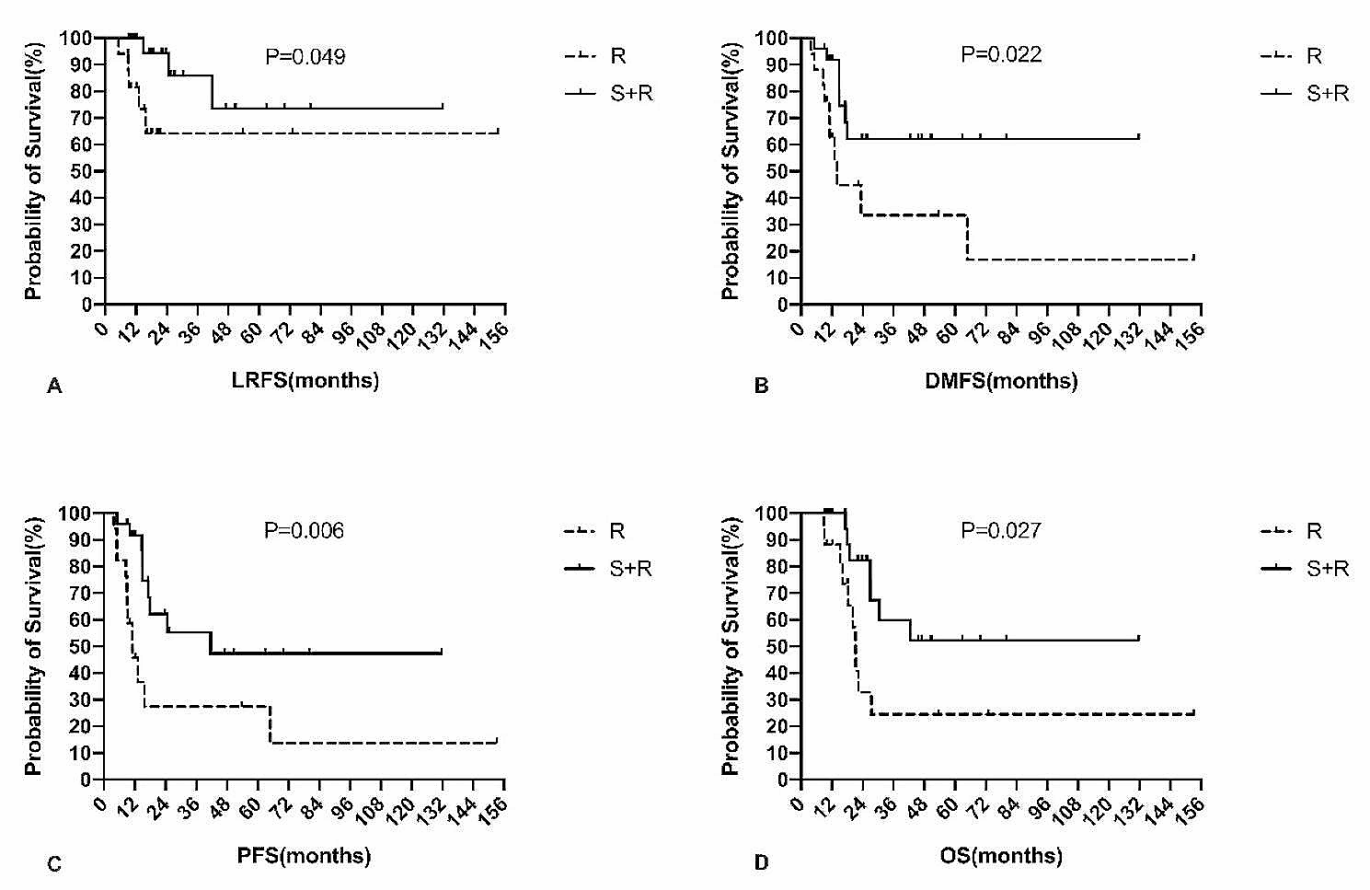
Local treatment patterns correlate with LRFS, DMFS, PFS and OS Abbreviations: LRFS, locoregional recurrence-free survival; PFS, progression-free survival; DMFS, distant metastasis-free survival; OS, overall survival

The 5-year locoregional recurrence-free survival (LRFS), progression-free survival (PFS), and OS rates of all patients were 69.0%, 31.7%, and 41.0%, respectively (Fig. 2). The median PFS and OS of all patients were 17.9 and 27.4 months, respectively. The effects of various demographic, clinical, and treatment-related variables on survival are summarised in Table 4. In this study, local treatment patterns (surgery and RT vs. RT alone) were significantly correlated with LRFS, distant metastasis-free survival (DMFS), PFS, and OS (Fig. 3).

**Table 4:** Univariate analysis of 42 patients with adult HNRMS

Prognostic Factor	5y-LRFS (%)	*P* value	5y-OS (%)	*P* value
Age		0.601		0.926
<28 yr	82.2		41.1	
≥ 28 yr	56.8		41.4	
Gender		0.825		0.318
Male	71.7		34.2	
Female	64.8		50.1	
Histology		0.276		0.267
Embryonal	57.3		27.3	
Alveolar	81.4		64.3	
Primary site		0.385		0.421
Parameningeal	58.9		36.5	
Non- Parameningeal	85.2		48.9	
Primary tumor size		0.047		0.142
≤ 5 cm	94.7		58.0	
> 5 cm	50.0		30.9	
Nodal status		0.029		0.478
N0	85.0		44.0	
N1	50.0		34.7	
Disease status		0.908		0.205
Local-regional (M0)	69.8		45.0	
Distant (M1)	Not reached		0	
IRS pretreatment staging		0.766		0.205
Stage I + II	78.4		53.6	
Stage III + IV	59.9		32.8	
IRS postsurgical grouping		0.385		0.333
Group I + II	65.3		43.7	
Group III + IV	71.9		38.3	
Surgery margins		0.162		0.301
Negative (R0)	100.0		83.3	
Positive (R1/2)	58.4		40.9	
Responses to chemotherapy		0.922		0.692
CR + PR	55.0		25.7	
SD + PD	70.5		44.3	
Chemoradiotherapy types		0.770		0.896
CCRT	67.1		37.0	
No-CCRT	76.2		42.9	
Local treatment patterns		0.049		0.027
Surgery + RT	73.6		52.4	
RT	64.2		24.5	

HNRMS, head and neck rhabdomyosarcoma; Intergroup Rhabdomyosarcoma Study, IRS; CR, complete response; PR, partial response; SD, stable disease; PD, progressive disease; LRFS, locoregional recurrence-free survival; OS, overall survival; CCRT, concurrent chemoradiation

**Fig. 4:**
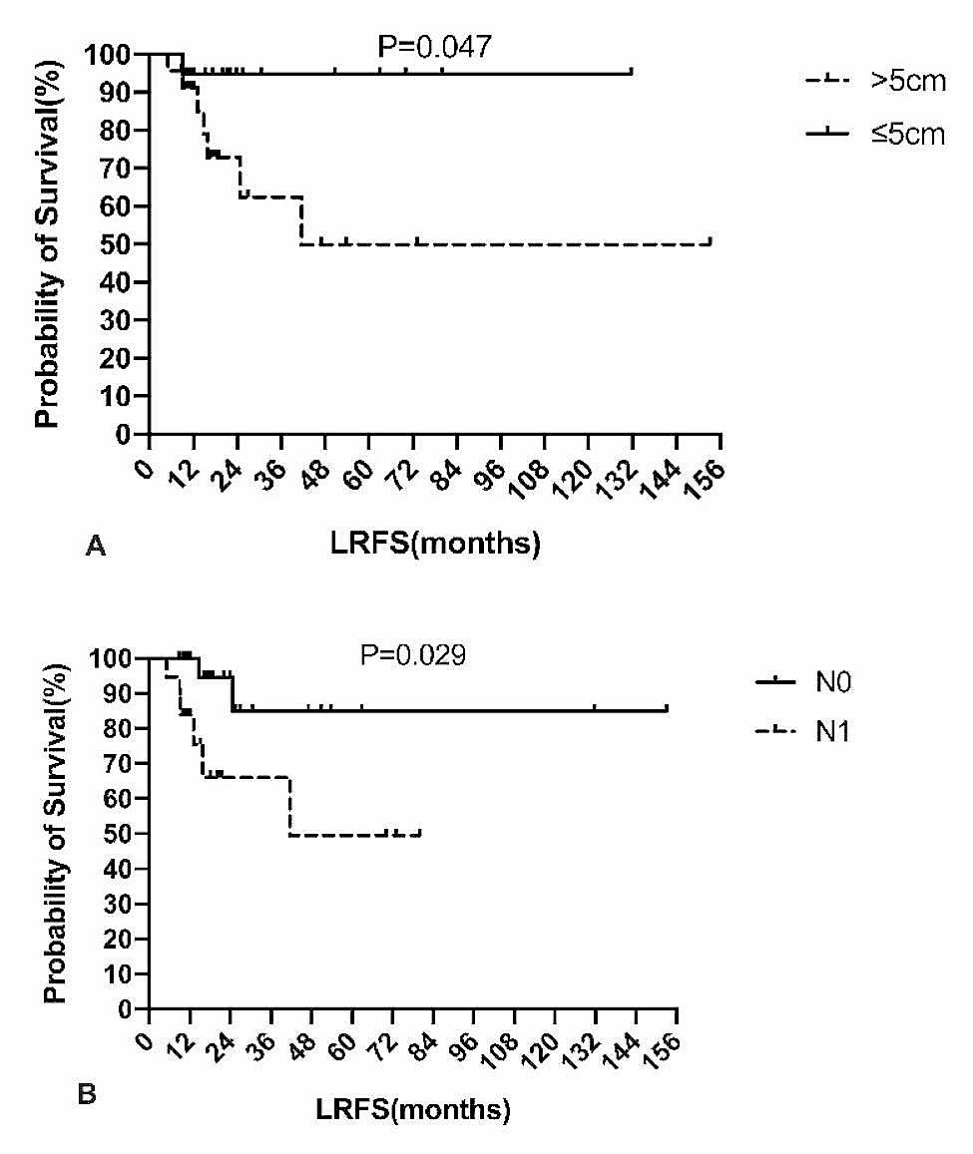
Tumor size and lymph node involvement correlated with LRFS Abbreviations: LRFS, locoregional recurrence-free survival

The results of univariate Cox proportional models demonstrated that margin status, chemoradiotherapy type, and response to chemotherapy were not prognostic factors for either OS or PFS in patients. In univariate analysis, increased tumour size and lymph node involvement were associated with worse LRFS (Fig. 4). The LRFS of the alveolar type seemed to be better than that of the embryonic type (5-year LRFS rate: 81.4% vs. 57.3%); however, the difference was not statistically significant (*P* = 0.276). Age, histopathological subtype, primary site, IRSG pretreatment staging, IRSG postsurgical grouping, and margin status did not affect LRFS outcomes.

## Discussion

HNRMS in adults is rare. There is a paucity of literature regarding the management and prognosis of adult patients with HNRMS, which mainly includes retrospective studies with small sample sizes or case reports [19, 20]. The current treatment guidelines for adult RMS are mainly based on the multimodality approach conceived by the IRSG, which aims to improve the long-term survival of paediatric RMS [21, 22]. Most studies have suggested that the prognosis of adult RMS is significantly worse than that of children. The 5year OS rate for adult RMS in our study was lower than that reported in paediatric studies [23–25] and similar to that reported in previous studies on adults with RMS [22, 26].

Two recent RMS studies included a large number of adults [1, 5]. In a recent SEER (Surveillance, Epidemiology, and End Results) database analysis of 1071 adults (> 19 years) with RMS [5], 100 patients were diagnosed with HNRMS. This study suggested that patients who received primary site-directed therapy in the form of surgery or RT had significantly better outcomes; however, no data regarding systemic therapy were available. Multivariate analysis revealed that age, tumour stage, and local treatment with surgery or radiation (or both) were significant predictors of adult survival. This finding was similar with our study. The estimated 5-year OS rate in this study was 27.0%, which was much lower than ours (41.0%). A recent series by Ferrari et al. [1], the largest retrospective single-institution study, evaluated 180 adult patients with RMS, of whom 109 received RT. Ferrari et al. reported 5-year event-free survival (EFS) and OS rates of 28.0% and 40.0%, which were lower than ours (39.7% and 41.0%). The ORR was 85.0% in patients with the embryonic and alveolar subtypes who were treated with chemotherapy, which was much higher than ours (51.8%). The EFS rate was 37.0% in patients who underwent complete resection, compared to 0% in patients with unresectable tumours.

In our study, we reviewed 42 adult patients with HNRMS to assess the efficacy of RT-based treatment and identify clinicopathological and treatment-related predictors. However, only local treatment patterns (Surgery + RT vs. RT) were independent predictors of OS. Male sex, parameningeal primary sites, embryonal subtype, increased tumour size, nodal involvement, IRS pretreatment stage III + IV, IRS postsurgical grouping III + IV and positive margins were associated with worse OS; however, these associations were not statistically significant. Univariate analysis showed that increased tumour size and lymph node involvement were associated with worse LRFS.

La Quaglia et al. [27] reviewed the effects of age on the outcomes of paediatric and adult RMS. Their findings showed that age at diagnosis was an independent predictor of the outcomes. Moreover, both the paediatric RMS trial by Joshi [28] and the retrospective trial on adult RMS by Iyad Sultan [5] showed that older age was associated with a poorer prognosis. Nevertheless, the true impact of age on survival remains controversial. Recent studies by Hahn et al. [2] and Wu et al. [29] have, however, shown that age is not a prognostic factor.

The effect of the patient’s sex on prognosis remains unclear in both paediatric and adult studies. Researchers have observed that the female sex is an adverse predictor of paediatric survival [30, 31]. However, in a retrospective analysis of a combined paediatric and adult RMS cohort from Memorial Sloan-Kettering [27], sex was not a prognostic factor. In our study, female patients were more likely to experience lymph node metastasis than male patients and showed a higher 5-year OS rate than male patients; however, the difference was not statistically significant in the univariate analysis. Recent studies on adult HNRMS by Hahn et al. [2] and Wu et al. [29] also found no significant association between sex and survival.

Previous studies have shown that embryonal and alveolar RMS subtypes are more common in childhood, whereas pleomorphic RMS occurs almost exclusively in adults [23, 32]. In this study, the incidence of pleomorphic HNRMS was extremely low (7.1%), which is inconsistent with the results of previous studies. It is well documented that children with embryonal histology have a better prognosis, whereas pleomorphic RMS is thought to be associated with a poor prognosis [33, 34]. However, our results showed an improved OS trend for patients with the alveolar subtype, with a 5-year OS rate of 64.3%, notably higher than that of patients with the embryonic subtype. This result may indicate a higher proportion of surgeries and a lower probability of lymph node involvement in patients with the alveolar subtype compared to those with the embryonic subtype. Therefore, the finding of improved survival in the alveolar subtype should be interpreted with caution. Due to the limited number of patients with the pleomorphic RMS subtype, data analysis was not possible.

The prognosis varies dramatically by primary site, with a 5-year OS rate of 89.0% for the orbit, 55.0% for non-orbital/non-parameningeal, and 47.0% for the parameningeal [35, 36]. Our results are similar to those reported in the literature. Statistical analysis indicated that primary tumour sites seem to influence prognosis, with 5-year OS rates of 48.9% and 36.5% for non-parameningeal and parameningeal tumours, respectively; however, these differences were not statistically significant. This could be attributed to all patients in our study receiving RT, which narrowed the gap in local recurrence rates between the two groups, resulting in statistically insignificant survival differences.

Tumour sizes > 5 cm are associated with worse prognoses in both paediatric and adult RMS [27]. Recent studies on adult HNRMS by Hahn et al. [2] and Wu et al. [29] also showed that increased tumour size was associated with worse OS. The role of lymph node involvement in prognosis remains controversial. Early research from La Quaglia et al. [27] showed that nodal involvement is a significant predictor of survival in both adult and paediatric patients with RMS. A review of 1,415 patients with IRS-I and IRS-II further confirmed that nodal involvement was an adverse prognostic factor [33]. Nevertheless, recent studies on adult HNRMS by Hahn et al. [2] and Wu et al. [29] did not find a correlation between nodal involvement and prognosis. In this study, both increased tumour size and lymph node involvement were associated with worse LRFS. However, these two factors did not correlate with OS, likely due to the limited sample size.

A study by Hawkins [23] confirmed that a positive margin indicated a poor prognosis for disease-specific survival in the adult population. Similarly, a recent study by Wu et al. [29] concluded that margin status was an independent prognostic factor for adult HNRMS. In their study, positive margins were associated with significantly poorer outcomes than negative margins, with 5-year OS rates of 6% and 50%, respectively. In our study, the margin status did not influence prognosis, possibly due to the compensatory effect of high-dose (66–70 Gy) RT in patients with positive margins after surgery.

In our study, the ORR to chemotherapy was 51.8%, which was lower than the rates reported in recent studies on adult HNRMS by Yang [37] and Wu [29], where ORRs reached 73.0% and 76.0%, respectively. However, despite a satisfactory response to chemotherapy in our study and in two other previous adult studies [23, 38], it did not effectively abrogate metastases, as 40.5% of patients in our study experienced distant metastasis. In our study, neither the response to chemotherapy nor concurrent chemoradiation emerged as a prognostic factor. The high metastatic rate of HNRMS necessitates ongoing investigation of various systemic therapies in adult HNRMS, such as multiagent chemotherapy, molecular targeting therapy, or immunotherapy.

A previous study by Hawkins demonstrated that all patients with local recurrences subsequently experienced distant failures [23]. In our experience, locoregional recurrence was accompanied or followed by distant failure in four out of eight cases (50.0%), indicating the importance of achieving local control. In our study, the local treatment methods included surgery and RT. Of the 42 patients, 25 received combined local treatment therapy (surgery and RT), whereas 17 received RT alone. A retrospective study from the MD Anderson Cancer Center involving 82 adults found that treatment choices (surgery + RT vs. RT alone) did not significantly correlate with OS or DFS [24]. However, our results indicated that the use of combined therapy significantly improved the 5-year locoregional control rate to 73.6% compared with 64.2% for RT alone (*P* = 0.049). Similarly, the choice of local treatment (surgery and RT vs. RT alone) significantly correlated with DMFS, PFS, and OS. These disparities may be attributed to the relatively lower radiation doses of 40–63 Gy administered to gross tumours at the MD Anderson Cancer Center, while our centre employed doses ranging from 66 to 70 Gy. Additionally, the earlier staging of patients in the combined local treatment therapy group may have played a role in these variations. These findings align with two previous randomised trials involving adult sarcomas, which also found that the addition of postoperative radiation resulted in significant improvements in local control [39, 40]. Therefore, we believe that optimal local control in adults can be achieved through a combination of maximal surgical resection and local postoperative RT at a relatively high dose.

We compared patients treated with Chemotherapy-Surgery-RadioChemotherapy (C-S) sequence to those with the Surgery-ChemoRadiotherapy (S-C) sequence, trying to understand whether exposure to early systemic therapy can influence the appearance of metastatic disease, which unfortunately has represented the major cause of death. In our study, 10 patients received C-S sequence, and 15 patients received S-C sequence. The 5-y LRFS, 5-y DMFS, and 5-y OS of C-S compared with S-C was 66.7% vs. 76.9% (*p* = 0.671), 80.0% vs. 55.2% (*p* = 0.287), and 37.5% vs. 56.4% (*p* = 0.674), respectively. It is difficult to answer the sequence and timing of treatments and their impact on long-term results by now, which need further larger sample study.

As for limitations, this was a retrospective study with a relatively small sample size, which may have restricted the data analysis. Despite this, we believe that our findings hold significance due to the limited availability of literature on HNRMS in adults.

## Conclusion

Adult HNRMS is a rare malignant tumour with a poor prognosis. Currently, no optimal treatment exists for adult HNRMS, mainly due to the limited number of studies on this infrequent group. Given that distant metastasis was the primary cause of treatment failure, the study highlights the need for careful monitoring and management of metastatic disease in adult HNRMS patients. This may prompt further research into systemic therapies and surveillance strategies. The study identifies key prognostic factors such as tumour size, lymph node involvement, and the local treatment pattern. These findings allow for better risk stratification of adult HNRMS patients and enable clinicians to identify high-risk patients who may require more aggressive treatment approaches. Although a standardised treatment remains undefined, localised HNRMS should be actively treated with multimodal approaches comprising surgery, RT, and systemic chemotherapy.

## Data Availability

The data are available from the corresponding author on reasonable request.

## Abbreviations

RMS: Rhabdomyosarcomas
HNRMS: Head and neck rhabdomyosarcomas
STS: Soft tissue sarcomas
IRSG: Intergroup Rhabdomyosarcoma Study Group
RT: Radiotherapy
IMRT: Intensity-modulated radiation therapy
CT: Computed tomography
MRI: Magnetic resonance imaging
ENI: Elective nodal irradiation
EFS: Event-free survival
IRS: Intergroup rhabdomyosarcoma studies
LRFS: Locoregional recurrence- free survival
PFS: Progression-free survival
OS: Overall survival.
